# Fabrication of Silk-Hyaluronan Composite as a Potential Scaffold for Tissue Repair

**DOI:** 10.3389/fbioe.2020.578988

**Published:** 2020-12-11

**Authors:** Li-Min Yu, Tao Liu, Yu-Long Ma, Feng Zhang, Yong-Can Huang, Zhi-Hai Fan

**Affiliations:** ^1^Shenzhen Key Laboratory of Spine Surgery, Department of Spine Surgery, Peking University Shenzhen Hospital, Shenzhen, China; ^2^Department of Textile Engineering, College of Textile and Clothing Engineering, Soochow University, Suzhou, China; ^3^Shenzhen Engineering Laboratory of Orthopaedic Regenerative Technologies, National and Local Joint Engineering Research Center of Orthopaedic Biomaterials, Peking University Shenzhen Hospital, Shenzhen, China; ^4^Department of Orthopedics, The Second Affiliated Hospital of Soochow University, Suzhou, China

**Keywords:** silk, hyaluronic acid, mesenchymal stem cells, biomimetic scaffold, tissue repair

## Abstract

Interest is rapidly growing in the design and preparation of bioactive scaffolds, mimicking the biochemical composition and physical microstructure for tissue repair. In this study, a biomimetic biomaterial with nanofibrous architecture composed of silk fibroin and hyaluronic acid (HA) was prepared. Silk fibroin nanofiber was firstly assembled in water and then used as the nanostructural cue; after blending with hyaluronan (silk:HA = 10:1) and the process of freeze-drying, the resulting composite scaffolds exhibited a desirable 3D porous structure and specific nanofiber features. These scaffolds were very porous with the porosity up to 99%. The mean compressive modulus of silk-HA scaffolds with HA MW of 0.6, 1.6, and 2.6 × 10^6^ Da was about 28.3, 30.2, and 29.8 kPa, respectively, all these values were much higher than that of pure silk scaffold (27.5 kPa). This scaffold showed good biocompatibility with bone marrow mesenchymal stem cells, and it enhanced the cellular proliferation significantly when compared with the plain silk fibroin. Collectively, the silk-hyaluronan composite scaffold with a nanofibrous structure and good biocompatibility was successfully prepared, which deserved further exploration as a biomimetic platform for mesenchymal stem cell-based therapy for tissue repair.

## Introduction

The combination of biomaterials and grafted cells with or without signalizing molecules has been regarded as a promising strategy for tissue repair, with the goal to provide structural and functional substitutes (Li et al., 2005; Ma et al., 2018; Qi et al., 2018). For successful repair, the scaffold should be elegantly designed to meet the requirements of damaged tissue (Raghunath et al., 2007). The characterization of the native extracellular matrix (ECM) in tissue, such as cartilage, is a network with multi-fibrillar collagens embedded in glycosaminoglycan (Zhang et al., 2011); the fibrous protein structure in ECM (50–500 nm) is about 1–2 orders of magnitude smaller than the cells, which is critical for cellular function (Woo et al., 2003). Thus, the scaffolds play a key role in the creation of the microenvironment for cell growth and tissue repair *in vitro* and *in vivo* (Dashnyam et al., 2014; Mahapatra et al., 2016). Thus, to mimic the ECM structure, the scaffolds with protein and glycosaminoglycan bi-components and nanofibrous structures are desired, aiming to control the adhesion, proliferation, migration, and differentiation of endogenous and exogenous progenitors/stem cells and then to moderate the tissue repair and reorganization *in vivo* (Chen and Ma, 2004; Ma, 2004; Toloue et al., 2019).

Because of the remarkable mechanical property, good biocompatibility, and biodegradability, silk is a very promising and encouraging material for the fabrication of tissue engineering scaffolds (Melke et al., 2016; Bhattacharjee et al., 2017). Over the past decades, silk has been processed into various formulations (such as film, nanofiber, hydrogel, fiber, yarn, and sponge) which have the potential to support the adhesion, proliferation, and differentiation of cells *in vitro* and to promote tissue regeneration *in vivo* (Wang et al., 2006; Rockwood et al., 2011). During the design of biomaterial from silk fibroin for tissue repair, 3D porous scaffold is the more promising material form as it provides the necessary 3-dimensional space for cell proliferation and new matrix formation (Wang et al., 2005); importantly, the particular requirements of composite scaffolds with respect to microporosity and nanostructure, as well as physical and biochemical properties, should be well considered. Silk has been processed into nanofibers to mimic the structure of collagen in the cartilage (Liu et al., 2016). Recently, we reported a method to prepare silk nanofiber by controlling the assembly process of silk fibroin and finally obtained the scaffold with an improved 3D porous structure and nanoscale topography (Zhang et al., 2018). Nevertheless, the pure silk lacks bioactive components to interact with the cell receptors for the activation of the tissue repair process (Garcia-Fuentes et al., 2009).

Hyaluronic acid (HA) is a natural biomaterial, and it has been developed for the application in the repair of ligament, adipose, bone, and cartilage (Abbruzzese et al., 2017). HA scaffold with bone marrow aspirate concentrate has been used to treat articular cartilage injury, displaying positive long-term clinical outcomes (Gobbi and Whyte, 2019). In spite of the promising results, pure HA scaffold has disadvantages such as the repaid degradation and the inadequate mechanical property (Yu et al., 2020).

During the process of fabricating functional silk scaffold, blending with high-molecular weight biocompatible polymers can be used to change the pore structure, porosity, microstructure, and mechanical properties; these important characteristics play a significant role in regulating the biology of grafted and resident cells (Jin et al., 2005; Lu et al., 2011). Silk has been blended with HA to mimic the ECM composition of cartilage (Jaipaew et al., 2016; Raia et al., 2017; Yan et al., 2018). The biological properties of HA are closely related to the molecular weight, and the high molecular weight HA displays superior biological and physical benefits. The superior biological properties are likely due to the high molecular weight of HA in animals; for example, in human synovial fluid, the molecular weight is 2,000–10,000 kDa (Ghosh and Guidolin, 2002). It has been found that chondrocyte number and matrix synthesis on gelatin sponge increased in the presence of high molecular weight HA (Goodstone et al., 2004). Our previous study demonstrated that HA interacted with silk in aqueous solution, resulting in the excellent porous structure of the composite scaffold, and the porous structure was dependent on the molecular weight of HA (Fan et al., 2014). The prepared composite has the ECM composition, but the nanofibrous structure is absent which is extremely essential in the mammal tissues (Ding et al., 2017).

Hence, in this study, a green and facile process to prepare silk-HA composite scaffold with biomimetic nanoscaled structure was presented. To achieve the goal, the following steps are involved: (1) self-assembly control of silk into nanofiber; (2) blending of silk nanofiber solution with HA with different molecular weights; and (3) lyophilization to form the composite scaffold. Additionally, the biocompatibility was evaluated using bone marrow mesenchymal stem cells *in vitro*.

## Materials and Methods

### Materials

Raw silk from *B. mori* was purchased from Jiangsu Silk Industrial Co., Ltd. (Nanjing, China). Na_2_CO_3_ and LiBr were from Sinopharm Chemical Reagent (Shanghai, China). HA (MW, 0.6, 1.6, and 2.6 × 10^6^ Da) was purchased from Shandong Freda (Jinan, China). All reagents for the cell culture were purchased from Invitrogen (Basel, Switzerland).

### Preparation of the Silk Solution

A silk solution was prepared as described in our previous study (Zhang et al., 2018); in brief, the procedures included degumming with Na_2_CO_3_, dissolving in LiBr solution, and dialysis using a dialysis tube. The resulting silk solution was optically clear and was centrifuged to remove aggregates. Determined by weighing, the concentration of the silk solution was approximately 6 wt.%. The fresh silk solution was then concentrated to 30 wt.% in an oven at 60°C and then diluted to 5 wt.% solution. The diluted silk solution was lyophilized to form porous scaffold for further use.

### Preparation of the Silk-HA Scaffold

As described in our previous study, HA was dissolved in deionized water to form 0.5 wt.% HA solution with the MW of 0.6, 1.6, and 2.6 × 10^6^ Da, respectively (Fan et al., 2014). The HA solutions were blended with the obtained 5 wt.% silk solution with the ratio of 1:1 at room temperature for 2 h. Finally, the mixed solution containing 2.5 wt.% silk and 0.25 wt.% HA was frozen at −20°C for about 24 h and then lyophilized for about 72 h. The lyophilized silk and silk-HA scaffolds were placed on a removable platform under which 75% ethanol was filled in a desiccator with a 25 in. Hg vacuum for 12 h to induce silk crystallization.

### Swelling

Silk and silk-HA scaffolds were immersed in distilled water at 37°C for 50 h. After removing the excess water, the weight of the wet scaffold (W_w_) was determined; after drying, the weight of dry scaffolds (W_d_) was determined again. The swelling ratio and water uptake of scaffolds were calculated as follows:

Swelling ratio = (W_w_ − W_d_)/W_d_
Water uptake (%) = (W_w_ − W_d_)/W_w_ × 100

### Biodegradation

Silk and silk-HA scaffolds (dry weight approximately 50 mg) (*n* = 3 per group and time points) were incubated at 37°C in 50 ml PBS (pH = 7.4) for 30 days. Samples were rinsed in distilled water and lyophilized for SEM and degradation evaluation at designated time points.

### Scanning Electron Microscopy (SEM) Analysis

Silk and silk-HA scaffolds were cut with a razor blade in liquid nitrogen. The cross-section was gold-sputtered and then observed with SEM (Hitachi S-4800, Tokyo, Japan).

### Fourier Transform Infrared Spectroscopy (FTIR)

FTIR spectra of the silk-HA scaffolds were conducted using a NicoLET 5700 spectrometer (Thermo Fisher Scientific, Waltham, MA, United States) with the spectral region of 400–4,000 cm^–1^, and 64 scans coded with a resolution of 4 cm^–1^.

### X-Ray Diffraction (XRD)

The crystal structure of silk scaffolds was analyzed using XRD (X’PERT-Pro MPD, PANalytical Company, Netherlands), which was operated at 30 mA tube current and 40 kV tube voltage with diffraction angles 2°–45°, and the scanning speed was 2°/min.

### Mechanical Property

The compressive property of the four scaffolds in wet (10 mm in diameter and 10 mm in height) (*N* = 5 for each group) were measured using the Instron 3365 testing frame (Instron, Norwood, MA) with a 500 N loading cell according to the published method (Hu et al., 2019).

### Isolation of Bone Marrow Mesenchymal Stem Cells

Bone marrow mesenchymal stem cells (BMSCs) were isolated from SD rats according to our previous report (Zhang et al., 2015). All procedures of the animal experiments in this study were performed in accordance with Soochow University Guidelines for the Welfare of Animals. Rat BMSCs at passage five were seeded in the silk scaffolds with the density of 1.0 × 10^5^ cells per sample.

### Cell Morphology

The cellular morphology on the scaffolds was observed by SEM. The sample was prepared according to our previous introduction (Zhang et al., 2018). Briefly, the cell-loaded scaffolds were rinsed with PBS, fixed in 4% paraformaldehyde, dehydrated with a gradient of alcohol (50, 70, 80, 90, 100, 100%), and then lyophilized. After being coated with gold, the samples were examined with SEM at the voltage of 10 kV. Several different areas of the specimens were randomly examined using a Hitachi model S-4800 scanning electron microscopy (Hitachi, Tokyo, Japan).

### Cell Proliferation

To determine the cell proliferation in the scaffolds, the samples harvested at the indicated time points (from 1 to 16 days) were digested with proteinase K buffer solution for 16 h at 56°C (Zhang et al., 2018); the CCK-8 assay was then used to detect the viability and proliferation of BMSCs. Twenty microliter of CCK-8 plus 500 μl DMEM was replaced to each well for 4 h at 37°C; subsequently, the 300 μl supernatant per well was transferred to a new 96-well plate and the absorbance value was measured using a microplate reader at 450 nm.

### Statistical Analysis

All experiments were carried out in triplicate, and the data were expressed as means and standard deviation (SD); the data were analyzed using the one way or two-way ANOVA followed by LSD multiple-comparison tests with the SPSS 19.0 software (IBM Corp., Armonk, NY, United States). The significance was accepted when *p* < 0.05.

## Results and Discussion

### Morphology of Silk-HA Scaffolds

**FIGURE 1:**
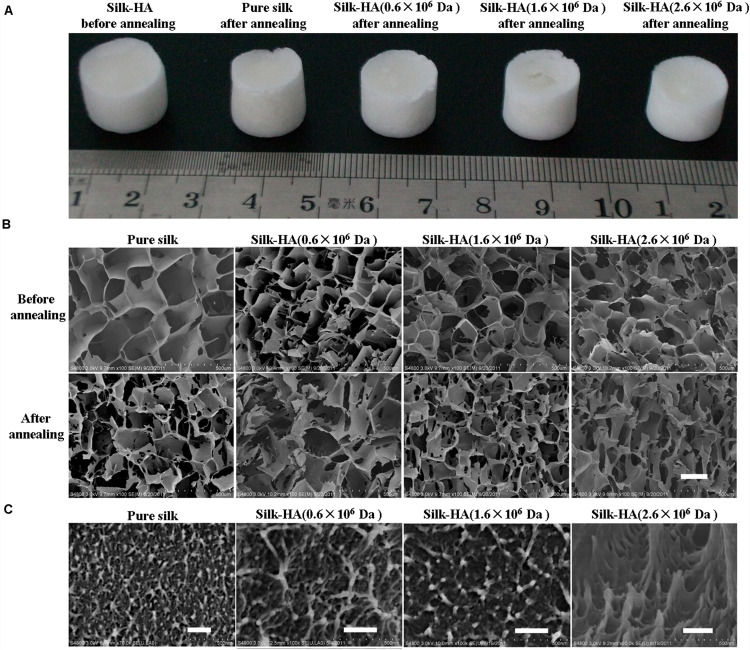
Gross view and microstructure of the silk-HA scaffolds. **(A)** Gross view of the pure silk and silk-HA scaffolds before and after 75% ethanol annealing. **(B)** SEM images of silk-HA scaffolds before and after 75% ethanol annealing. **(C)** The cross-section images of macropore walls of silk-HA scaffolds. Scale bar = 200 μm.

Silk and silk-HA porous scaffolds were prepared using 24-well plates with the freeze-drying method and then annealed with 75% ethanol. The appearance of silk-HA scaffolds is shown in Figure 1A. Overall, the silk-HA scaffolds displayed intact appearance, which was negligibly affected by 75% ethanol annealing. In our previous report (Fan et al., 2014), pure silk scaffold underwent serious morphologic changes, which was improved by adding HA with high molecular weight. In this work, the pure silk scaffold showed good ability to resist to shape deformation, likely due to the excellent porous structure within the scaffolds. As previously reported, the self-assembled nanofilament had an important role in forming good pore structure for lyophilized silk scaffold (Lu et al., 2011).

The porous structure of silk and silk-HA scaffolds before and after 75% ethanol annealing was observed by SEM, as shown in Figure 1B. The scaffolds were fabricated from silk-HA blend solutions containing about 10% HA with the MW of 0.6 × 10^6^, 1.6 × 10^6^, and 2.6 × 10^6^ Da, respectively. As expected, the nice pore structure instead of the lamellar structure was formed from pure silk solution, indicating that the silk nanofiber was able to induce the formation of a porous structure and restrain the lamellar structure. It was demonstrated that the silk scaffold derived from fresh solution usually showed separate lamellas rather than a porous structure which was not suitable for the application for tissue repair (Lv and Feng, 2006). The porous architecture was critical for the biomedical scaffold so as to allow both the cellular ingrowth and vascularization, which was essential for new tissue formation (Bonfield, 2006). The effect of HA MW on the porous structure of silk-HA scaffold could be negligible. As noted in previous reports (Lu et al., 2010, 2011), the blending of biocompatible polymer collagen and gelatin favored the formation of porous structures due to the molecular interaction between silk and polymers. Additionally, after closer examination by SEM, the nanoscaled topographies were clearly found in the pore wall (Figure 1C). The ECM-mimetic nanofibrous structure was able to improve the cell adhesion and proliferation (Liu et al., 2014). The porosity of the silk-HA scaffolds was measured by liquid displacement (Hu et al., 2019). The structure of the silk-HA scaffolds was very porous, with the porosity of 99 ± 5, 98 ± 4, and 98 ± 3% when the HA MW was 0.6 × 10^6^, 1.6 × 10^6^, and 2.6 × 10^6^ Da, respectively; these values were similar to that of pure silk scaffolds (99 ± 4%). Thus, pure silk and silk-HA scaffolds with excellent porous structures and specific nanostructure could be achieved directly by lyophilizing the silk and silk-HA blending solutions.

### Swelling

**FIGURE 2:**
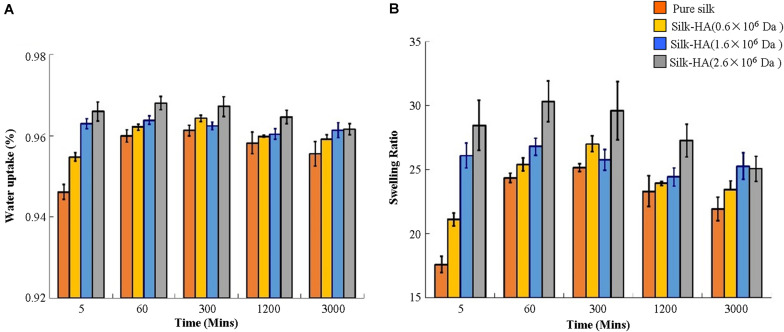
Change in the water uptake **(A)** and swelling ratio **(B)** of silk-HA scaffolds with time.

Compared with pure silk scaffolds, the water uptake and the swelling ratio were increased significantly with the increase of molecular weight of HA in these scaffolds (Figure 2). It is believed that as the MW increases, the resulting material after swelling will have a larger mesh size, resulting in a higher swelling ratio (Park et al., 2009). A previous study has reported that the HA 1,368 kDa film possessed a significantly higher swelling ratio than HA 1,058 and 697 kDa films (Lee et al., 2015). The swelling ratio of scaffolds reached their maximum about 60 min, and then the values were gradually decreased. The data indicated that the swelling ratio of the silk-HA scaffold could be adjusted by changing the initial HA molecular ratio.

### *In vitro* Degradation

**FIGURE 3:**
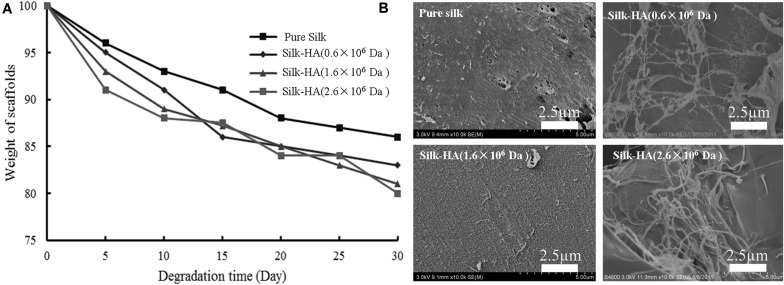
Degradation of silk-HA scaffolds in PBS **(A)** and the SEM images **(B)** of silk-HA scaffolds degraded in PBS solution at 37°C for 30 days. Scale bar = 2.5 μm.

*In vitro* degradation of silk-HA scaffolds was evaluated by incubation with PBS solution (Lu et al., 2011). The weight of silk-HA (1.6 × 10^6^ and 2.6 × 10^6^ Da) scaffolds decreased slowly with time in PBS solution; they lost 12% mass after 15 days and then 20% mass after 30 days (Figure 3A). The morphology change after degradation was observed by SEM, indicating that the silk-HA scaffolds degraded in the mean of pore damage and surface erosion (Figure 3B); meanwhile, the nanofibers were found on the surface of the macro-pore walls, which was in agreement with the previous reports (Lu et al., 2011; Pei et al., 2015; Zhang et al., 2018). These data indicated that the self-assembly silk nanofilament could endow the resulting silk-HA scaffolds with nanofibrous structure exposure after degradation. Hence, the silk-HA scaffolds with nanofibrous structure and biomimetic content of silk and HA were successfully fabricated.

### Structural Analysis

**FIGURE 4:**
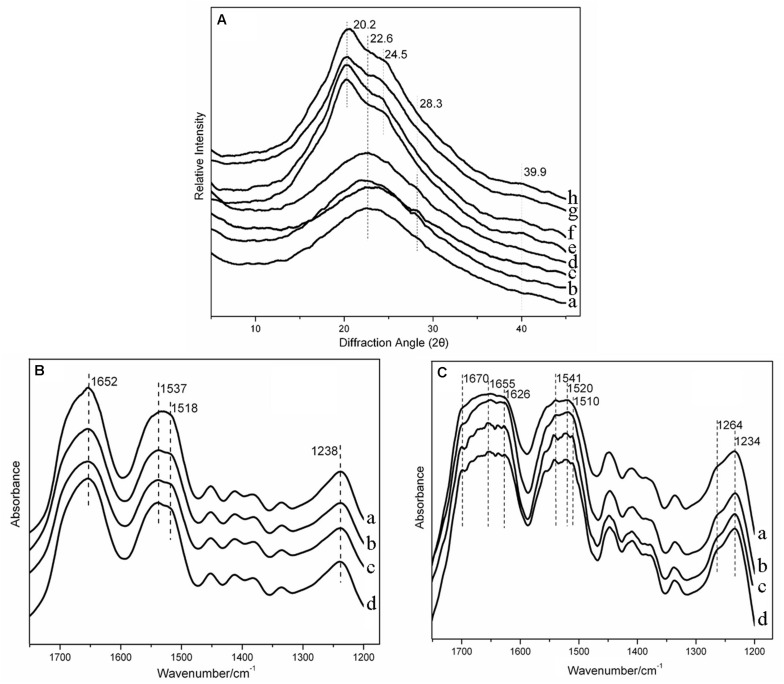
XRD spectra of silk-HA scaffolds **(A)**. (a–d) Pure silk scaffold, silk-HA scaffolds with HA MW 0.6 × 10^6^, 1.6 × 10^6^, and 2.6 × 10^6^ Da before 75% ethanol annealing. (e–h) Pure silk scaffold, silk-HA scaffolds with HA MW 0.6 × 10^6^, 1.6 × 10^6^, and 26 × 10^6^ Da after 75% ethanol annealing. FTIR spectra of silk-HA scaffolds before **(B)** and after **(C)** 75% ethanol annealing. (a) Pure silk scaffold. (b–d) Silk-HA scaffolds with HA MW 0.6 × 10^6^, 1.6 × 10^6^, and 2.6 × 10^6^ Da before 75% ethanol annealing.

The XRD patterns of silk fibroin had been determined in the previous study as follows: 9.0°, 18.5°, and 20.6° for the silk II structure, and 12.0°, 15.8°, 20.2°, 21.6°, 24.7°, 27.8°, and 31.9° for the silk I structure (Zhang et al., 2018). Figure 4A pointed out the crystal structure in the silk-HA scaffolds. The untreated scaffolds were mainly amorphous in structure, characterized by a broad amorphous halo centered around 22° (Phillips et al., 2004). The weak diffraction peak at 28.3° in the HA-contained scaffold suggested the existence of silk I which was probably induced by HA. After annealing, the structural transition to the insoluble crystal structure was achieved, characterized by the specific silk II peak at 20.2° and silk I peak at 24.5°. The FTIR characterization was conducted to further confirm the XRD results, as shown in Figures 4B,C. The pure silk scaffold before 75% ethanol annealing showed absorption peaks at 1,652, 1,537, and 1,238 cm^–1^, which was in accordance with the amorphous structure (Jin et al., 2005). The absorption peak at 1518 cm^–1^ existed in the silk-HA scaffolds, indicating the role of HA in inducing the conformation transition of silk (Chen et al., 1997). After annealing (Figure 4C), the silk-HA scaffolds had absorption peaks at 1,670, 1,626, 1,520, 1,510, and 1,264 cm^–1^, suggesting that their major structure was silk II (Hu et al., 2006). Thus, the FTIR and XRD results jointly confirmed that the silk-HA scaffold after annealing mainly had the water-stable crystal structure.

### Mechanical Property

**FIGURE 5:**
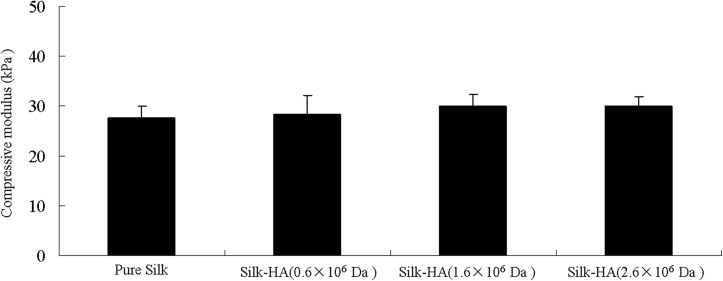
Compressive modulus of the silk-hyaluronan scaffolds in wet conditions. The compressive modulus was calculated as the slope of the linear-elastic region of the stress–strain curve between 3 and 8%.

The mechanical properties of silk-HA scaffolds are illustrated in Figure 5. The compressive modulus of the silk-HA scaffolds with HA MW 0.6 × 10^6^, 1.6 × 10^6^, and 2.6 × 10^6^ Da was about 28.3, 30.2, and 29.8 kPa, respectively; all these results were higher than that of pure silk scaffolds (27.5 kPa). It has been reported that the scaffold with stiffness of 25 kPa was able to promote proliferation and chondrogenic differentiation of MSCs (Zhan, 2020), suggesting that the stiffness control of the scaffold is important for cartilage tissue engineering. Thus, the prepared silk-HA scaffold with elastic modulus of 27.5–30.2 kPa probably has promising application in cartilage repair.

### Biocompatibility of Silk-HA Scaffolds

**FIGURE 6:**
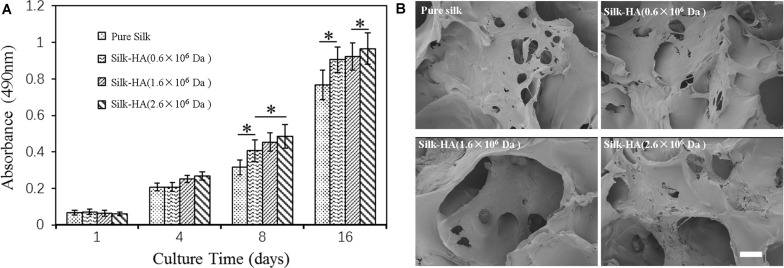
Proliferation **(A)** and morphology **(B)** of BMSCs in the pure silk and silk-HA scaffolds after culture for 16 days (*P < 0.05).

To assess the biocompatibility, BMSCs were cultured in the silk-HA scaffolds. Figure 6 shows the proliferation and morphology of BMSCs in the silk-HA scaffolds. The proliferation behavior of BMSCs in the scaffolds was evaluated by CCK8 (Figure 6A). The results indicated that the cells grew well in the four scaffolds and the cell number in the silk-HA scaffolds was much higher than that in the pure silk after culture for 8 and 16 days (*P* < 0.05); additionally, the silk-HA (2.6 × 10^6^ Da) scaffold possessed the highest absorbance value when compared with those of the other three groups (*P* < 0.05). As noted in Figure 6B, the BMSCs adhered and proliferated well in the scaffolds; on day 16, the increased cells formed a cell sheet and covered on the pore or pore wall of the scaffolds. The HA component has been known to promote cell migration and proliferation through receptors, such as CD44 (Garcia-Fuentes et al., 2009; Murakami et al., 2019). The silk-HA scaffold could provide a more suitable microenvironment of biomimetic composition and structure for cell proliferation (Lu et al., 2011; Ding et al., 2017). The biological effects of HA depended heavily on molecular weight (Snetkov et al., 2020). The molecular weight of HA in human synovial fluid is very high, with the range of 2,000–10,000 kDa (Ghosh and Guidolin, 2002). Previous studies have proved the biological and physical benefits of high molecular weight HA (Abe et al., 2005; Ohtsuki et al., 2018). Therefore, our data combined with previous literatures which demonstrated that the silk-HA composite scaffolds favored cell-biomaterial interactions and enhanced cell growth. Further studies are extremely necessary to address and optimize the effect of silk-HA scaffolds on the differentiation of BMSCs for repairing tissue defect *in vitro* and in animal studies.

## Conclusion

In this study, a friendly process for the preparation of silk-HA composite scaffolds with a nanofibrous structure for tissue repair was provided. A silk molecule was first assembled into silk nanofibers which endowed the resulting scaffolds with an ECM-mimetic structure. The mean porosity of these silk-HA scaffolds was up to 99%, and compressive modulus was about 29 kPa. These silk-HA scaffolds were biodegradable (up to 20% degradation rate in PBS); possessed good pore structure, suitable compositions, and biomimetic nanofibrous structure; and enhanced BMSC growth and proliferation. The biocompatibility, biodegradability, and specific nano-to-micro structure made these silk-HA composite materials as promising scaffolds for tissue repair (such as cartilage) which need further investigation.

## Data Availability Statement

The original contributions presented in the study are included in the article/supplementary materials, further inquiries can be directed to the corresponding author/s.

## Ethics Statement

The animal study was reviewed and approved by the Soochow University.

## Author Contributions

L-MY contributed to the experimental design, data interpretation, and manuscript writing. TL, Y-LM, and FZ performed the experiments, collected the data, and wrote the manuscript. Y-CH and Z-HF provided financial support, data analysis and interpretation, and manuscript writing. All the authors approved the final manuscript to be submitted.

## Conflict of Interest

The authors declare that the research was conducted in the absence of any commercial or financial relationships that could be construed as a potential conflict of interest.
